# Correction to: A model of Hill-Robertson interference caused by purifying selection in a nonrecombining genome

**DOI:** 10.1093/genetics/iyaf186

**Published:** 2025-10-06

This is a correction to: Hannes Becher, Brian Charlesworth, A model of Hill-Robertson interference caused by purifying selection in a nonrecombining genome, *Genetics*, Volume 230, Issue 1, May 2025, iyaf048, https://doi.org/10.1093/genetics/iyaf048

In the originally published version of this manuscript, the following sentence in the Methods section required correction: “The mutation rates for A_1_ to A_2_ and A_2_ to A_1_ are *κu* and *u*, respectively, where *κ i*s the mutational bias parameter.”

The sentence should read “The mutation rates for A_1_ to A_2_ and A_2_ to A_1_ are *u* = *κv* and *v*, respectively, where *κ i*s the mutational bias parameter.”

In Table 1, the values of *N_o_s* should have been 0.02, 2 and 200, not 0.2, 20 and 200.

These errors have been corrected.

